# Disguised as a Sulfate Reducer: Growth of the Deltaproteobacterium *Desulfurivibrio alkaliphilus* by Sulfide Oxidation with Nitrate

**DOI:** 10.1128/mBio.00671-17

**Published:** 2017-07-18

**Authors:** Casper Thorup, Andreas Schramm, Alyssa J. Findlay, Kai W. Finster, Lars Schreiber

**Affiliations:** a Center for Geomicrobiology, Aarhus University, Aarhus, Denmark; b Section for Microbiology, Department of Bioscience, Aarhus University, Aarhus, Denmark; c Department of Geological and Environmental Sciences, Ben-Gurion University of the Negev, Be’er Sheva, Israel; California Institute of Technology/HHMI

**Keywords:** DNRA, DSR, nitrate reduction, nitrite reduction, sulfate reduction, sulfide oxidation, sulfur cycle

## Abstract

This study demonstrates that the deltaproteobacterium *Desulfurivibrio alkaliphilus* can grow chemolithotrophically by coupling sulfide oxidation to the dissimilatory reduction of nitrate and nitrite to ammonium. Key genes of known sulfide oxidation pathways are absent from the genome of *D. alkaliphilus*. Instead, the genome contains all of the genes necessary for sulfate reduction, including a gene for a reductive-type dissimilatory bisulfite reductase (DSR). Despite this, growth by sulfate reduction was not observed. Transcriptomic analysis revealed a very high expression level of sulfate-reduction genes during growth by sulfide oxidation, while inhibition experiments with molybdate pointed to elemental sulfur/polysulfides as intermediates. Consequently, we propose that *D. alkaliphilus* initially oxidizes sulfide to elemental sulfur, which is then either disproportionated, or oxidized by a reversal of the sulfate reduction pathway. This is the first study providing evidence that a reductive-type DSR is involved in a sulfide oxidation pathway. Transcriptome sequencing further suggests that nitrate reduction to ammonium is performed by a novel type of periplasmic nitrate reductase and an unusual membrane-anchored nitrite reductase.

## IMPORTANCE

Sulfide oxidation and sulfate reduction, the two major branches of the sulfur cycle, are usually ascribed to distinct sets of microbes with distinct diagnostic genes. Here we show a more complex picture, as *D. alkaliphilus*, with the genomic setup of a sulfate reducer, grows by sulfide oxidation. The high expression of genes typically involved in the sulfate reduction pathway suggests that these genes, including the reductive-type dissimilatory bisulfite reductases, are also involved in as-yet-unresolved sulfide oxidation pathways. Finally, *D. alkaliphilus* is closely related to cable bacteria, which grow by electrogenic sulfide oxidation. Since there are no pure cultures of cable bacteria, *D. alkaliphilus* may represent an exciting model organism in which to study the physiology of this process.

## OBSERVATION

Sulfide is produced during the degradation of organic matter by fermenting and sulfate-reducing microorganisms (SRMs). Its high reduction potential makes it an attractive substrate for photo- and chemolithotrophic sulfide-oxidizing microorganisms (SOMs). SOMs are taxonomically diverse and can be found within the class *Chloroflexi* and the phyla *Chlorobi* and *Firmicutes*, as well as in the classes *Alphaproteobacteria*, *Betaproteobacteria*, and *Gammaproteobacteria* (1). Fuseler et al. (2) demonstrated that sulfate reducers belonging to the genera *Desulfobulbus* and *Desulfovibrio* are also able to oxidize sulfide by combining its oxidation with either oxygen or nitrates as electron acceptors with the disproportionation of intermediately produced elemental sulfur. The organisms tested were unable to couple the combined process to growth. Recently, the ability to couple chemolithotrophic sulfide oxidation with arsenate, nitrate, or oxygen reduction to growth has been reported for microorganisms belonging to the *Desulfobulbaceae* family (3, 4). However, the pathways of sulfide oxidation have not been elucidated yet.

Here we present a novel case of a deltaproteobacterium, *Desulfurivibrio alkaliphilus*, performing growth-coupled chemolithotrophic sulfide oxidation by dissimilatory nitrate and nitrite reduction to ammonium (DNRA). We used a comparative genomic and transcriptomic approach to obtain first insights into the species’ pathways of sulfide oxidation and DNRA.

### *D. alkaliphilus* grows by sulfide oxidation coupled with DNRA.

**FIG 1:**
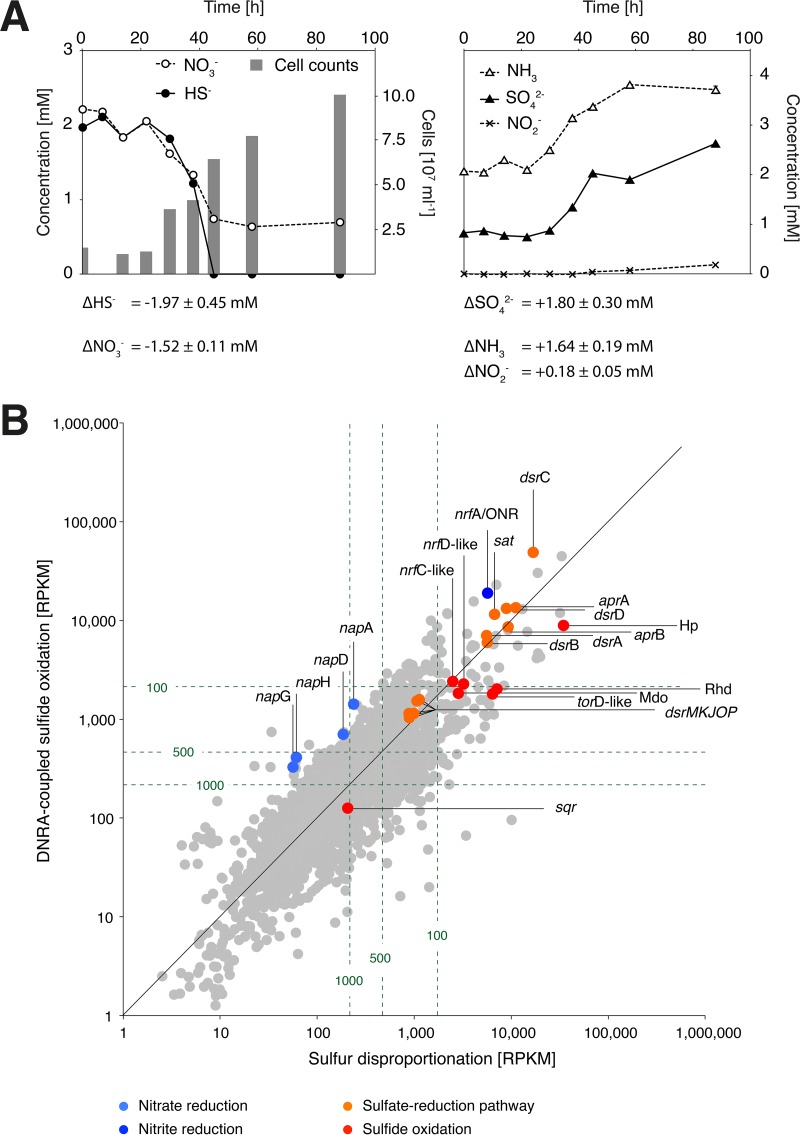
Growth curve, reagent concentrations, and gene expression. (A) Reagent concentrations and *D. alkaliphilus* cell numbers during growth by sulfide oxidation coupled with DNRA. Net changes in reagent concentrations over the course of the experiment and corresponding standard deviations are shown below the graphs. All values represent the mean of triplicate cultures. (B) Gene expression of *D. alkaliphilus* during growth by sulfide oxidation coupled with DNRA and by disproportionation of elemental sulfur. Genes are represented by dots and are positioned according to their RPKM expression values. Genes relevant to this study are color coded and annotated. Dashed lines indicate isopleths of RPKM ranks; e.g., genes to the right of the dashed 100 line are among the 100 most expressed genes during growth by elemental sulfur disproportionation.

*D. alkaliphilus* is an alkaliphilic bacterium within the family *Desulfobulbaceae* (class *Deltaproteobacteria*), which is composed primarily of strictly anaerobic sulfate reducers. Originally, *D. alkaliphilus* was reported to grow by coupling the oxidation of short-chain fatty acids and H_2_ to the reduction of sulfur compounds or nitrate (5). Recently, Poser et al. (6) demonstrated that *D. alkaliphilus* can grow by disproportionation of elemental sulfur. We show here for the first time that *D. alkaliphilus* grows by sulfide oxidation coupled with DNRA (Fig. 1A) with nitrate, as well as nitrite, as an electron acceptor (see Fig. S1A in the supplemental material). Growth by nitrate reduction yielded small concentrations of nitrite in late culture stages (Fig. 1A), indicating nitrite as an intermediate of DNRA. Substrate and product concentrations for DNRA-coupled sulfide oxidation by *D. alkaliphilus* agree with the following stoichiometry: $HS^{-}+NO_{3}^{-}+H_{2}O\;\to \;SO_{4}^{2-}+NH_{3}$.

10.1128/mBio.00671-17.1 FIG S1  Reduction of nitrate and nitrite by *D. alkaliphilus* (A) Increase in cell density of *D. alkaliphilus* during growth by sulfide oxidation coupled with the reduction of nitrate or nitrite. (B) Model of DNRA by *D. alkaliphilus*. The reconstruction is based on genes highly expressed or upregulated during growth of *D. alkaliphilus* by sulfide-dependent nitrate reduction (see also Table S3). Enzymes previously proposed to be involved in DNRA but whose genes were not detected in *D. alkaliphilus* are shaded in gray. NrfC*, the detected putative *nrfC* gene in the *D. alkaliphilus* genome does not feature the necessary signal peptide for periplasmic localization. Download FIG S1, PDF file, 0.2 MB. Copyright © 2017 Thorup et al. 2017 Thorup et al. This content is distributed under the terms of the Creative Commons Attribution 4.0 International license.

### **Sulfur metabolism of** *D. alkaliphilus*.

With the exception of a sulfide:quinone oxidoreductase (SQR), we could not detect any key genes of known microbial sulfide oxidation pathways (1), i.e., genes coding for flavocytochrome *c* or the SOX system, in the genome of *D. alkaliphilus*. Instead, the genome contains all of the key genes of the sulfate reduction pathway (7) (Table S1). Transcriptomic analysis showed high levels of expression of all of these genes during growth by sulfide oxidation (Fig. 1B). Especially genes coding for the four enzymes responsible for transforming sulfur compounds, namely, the sulfate adenylyltransferase (Sat), the adenosine-5′-phosphosulfate (APS) reductase (APR), the dissimilatory bisulfite reductase (DSR), and the DsrC protein, were among the most highly expressed genes (Fig. 1B).

10.1128/mBio.00671-17.6 TABLE S1  Presence and expression of *D. alkaliphilus* genes involved in sulfur metabolism. Locus tags for GenBank and the IMG database are listed. Gene expression is shown as RPKM ranks and as log_2_-fold changes (logFC). Abbreviations: sulfur, growth by elemental sulfur disproportionation conditions; nitrate, growth by DNRA-coupled sulfide oxidation. Download TABLE S1, PDF file, 0.2 MB. Copyright © 2017 Thorup et al. 2017 Thorup et al. This content is distributed under the terms of the Creative Commons Attribution 4.0 International license.

**FIG 2:**
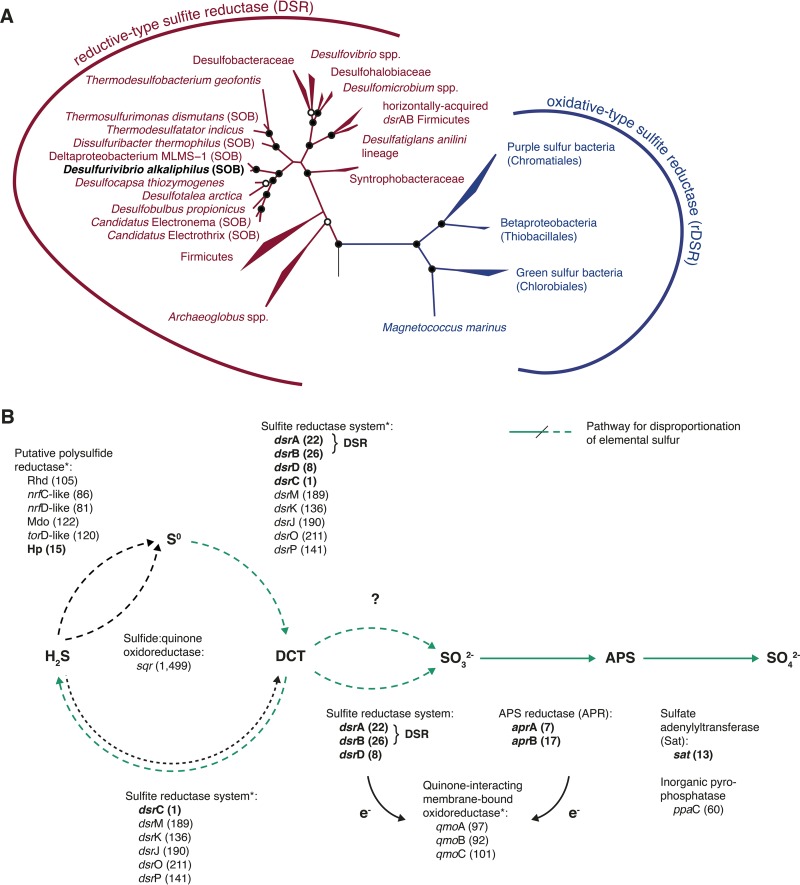
Phylogeny of sulfite reductases and proposed novel pathway of sulfide oxidation. (A) Phylogeny of oxidative- and reductive-type sulfite reductases based on maximum-likelihood analysis. Bootstrap support values (*n* = 1,000) are indicated by full (≥90%) or empty (≥70%) circles. Bacteria having a reductive-type sulfite reductase and growing by chemolithotrophic sulfide/sulfur oxidation are indicated by the abbreviation SOB. The tree was rooted with the archaeal reductive sulfite reductase of *Vulcanisaeta* spp. (not shown). (B) Proposed pathway of chemolithotrophic sulfide oxidation by *D. alkaliphilus*. Shown are the enzymes proposed to be involved and the associated genes. Gene expression rank during growth by sulfide oxidation is shown in parentheses. Highly expressed genes (within the top 30) are in boldface. The pathway for sulfur disproportionation is green. Asterisks indicate that electrons transferred to these membrane-associated enzyme complexes are used to reduce menaquinone and are presumably consumed by DNRA. Abbreviations used for the putative polysulfide reductase operon: Rhd, rhodanese; Mdo, molybdopterin oxidoreductase; Hp, hypothetical protein.

Recently, Santos et al. (7) demonstrated that sulfate reduction is a four-step process in which (i) the Sat enzyme activates the chemically stabile sulfate molecule forming APS with a very energetic sulfate-phosphate-anhydrate chemical bond; (ii) APS is reduced to bisulfite by APR; (iii) bisulfite is reduced to DsrC-trisulfide (DCT) by the DSR and DsrC with the concomitant oxidation of two conserved cysteine during trisulfide formation; and (iv) DCT is reduced by the DsrMKJOP complex, which is located at the cell membrane, a process during which the two conserved cysteines are reduced and hydrogen sulfide is released. Homologs of all four enzymes are also commonly encountered in SOMs (1). However, while Sat and APR are highly conserved between SRMs and SOMs (8, 9), reductively and oxidatively operating sulfite reductases, i.e., DSR and rDSR, as well as the reductively and oxidatively operating protein DsrC, form phylogenetically distinct clades (10). Surprisingly, in *D. alkaliphilus*, the *dsrA* and *dsrB* genes encoding the α and β subunits of the sulfite reductase affiliated with the DSR clade and are phylogenetically closely related to genes of other (sulfate-reducing) members of the family *Desulfobulbaceae* (Fig. 2A). Furthermore, the sulfite reductase of *D. alkaliphilus* has all of the characteristics of a functional reductive-type enzyme (Fig. S2). Similarly, the *dsrC* gene is also more closely related to those of SRMs than to those of SOMs (Fig. S3). Lastly, in SOMs, the *dsrEFH* operon is essential for the function of rDSRs (11). The *dsrEFH* operon is missing from the genome of *D. alkaliphilus*. Overall, *D. alkaliphilus* shows the genomic makeup of SRMs and cannot be distinguished from them on the basis of genomic features alone.

10.1128/mBio.00671-17.2 FIG S2  DSR of *D. alkaliphilus* compared to selected DSRs and rDSRs. Conserved amino acids are shaded blue (DsrA subunit) or magenta (DsrB). The degree of conservation is indicated by dark (strongly conserved) or light (moderately conserved) shading. Residues involved in sulfite transport and binding (T. F. Oliveira, E. Franklin, J. P. Afonso, A. R. Khan, N. J. Oldham, I. A. Pereira, and M. Archer, Front Microbiol 2:71, 2011, https://doi.org/10.3389/fmicb.2011.00071) are marked as follows: circles, strictly conserved residues; triangles, additionally involved residues. Essential siroheme binding motifs are indicated. The alignment was visualized with BOXSHADE version 3.21 (http://sourceforge.net/projects/boxshade). Download FIG S2, PDF file, 0.3 MB. Copyright © 2017 Thorup et al. 2017 Thorup et al. This content is distributed under the terms of the Creative Commons Attribution 4.0 International license.

10.1128/mBio.00671-17.3 FIG S3  Phylogeny of the *dsrC* gene of *D. alkaliphilus*. Shown is a maximum-likelihood phylogeny of *dsrC* genes of *D. alkaliphilus* and known sulfate-reducing and sulfide-oxidizing bacteria. Circles represent bootstrap support after 1,000 resamplings as follows: open, ≥70%; filled, ≥90%. Download FIG S3, PDF file, 0.2 MB. Copyright © 2017 Thorup et al. 2017 Thorup et al. This content is distributed under the terms of the Creative Commons Attribution 4.0 International license.

### Proposal of a novel pathway for chemolithotrophic sulfide oxidation.

There are three additional cases of deltaproteobacteria growing by oxidation of sulfur compounds. Strain MLMS-1 couples sulfide oxidation to arsenate reduction (3), *Dissulfuribacter thermophilus* oxidizes elemental sulfur with DNRA (12), and cable bacteria (“*Candidatus* Electronema” and “*Ca*. Electrothrix”) grow by coupling sulfide oxidation by using long-distance electron transport to oxygen or nitrate reduction (4). Similar to *D. alkaliphilus*, both strain MLMS-1 and *D. thermophilus* lack the key genes of SOMs and instead feature all of the key genes for sulfate reduction (12) (Table S2). Cable bacteria have not been genomically characterized but feature reductive-type sulfite reductases characteristic of SRMs (4). Finally, the distantly related species *Thermosulfurimonas dismutans* couples the oxidation of elemental sulfur to DNRA but also features an SRM gene set (Table S2). On the basis of these genomic data and the observation that all of the key genes for sulfate reduction are highly expressed by *D. alkaliphilus* during growth by sulfide oxidation (Fig. 1B), we propose a novel, DSR-mediated pathway for sulfide oxidation, with reductive-type Dsr proteins as key elements (Fig. 2B). One possible scenario is the reversal of the pathway proposed by Santos et al. (7) and begins by DsrC directly interacting with sulfide to form the oxidized key intermediate DCT. However, neither *D. thermophilus* nor *T. dismutans*, which both possess the genes of the complete sulfate reduction pathway, including DsrC, can grow by sulfide oxidation, but both require elemental sulfur as an electron donor (12). This incapacity indicates that DsrC alone may not be sufficient for the initial sulfide oxidation. The DSR-mediated sulfide oxidation pathway would then start with the initial formation of elemental sulfur (Fig. 2B), as originally proposed for sulfide oxidation by SRMs (2). The initial oxidation of sulfide to sulfur without the involvement of DsrC would bypass the membrane-bound DsrMKJOP complex that donates electrons to DCT and regenerates DsrC. Nevertheless, all of the genes involved in this complex were highly expressed in our study. This leaves us with a puzzle concerning the initial oxidation of sulfide, which we have not conclusively resolved yet. Interestingly, Fuseler et al. (2) could not measure sulfite reductase activity, which is in contrast to the high level of expression of all of the Dsr-encoding genes that we observed. On the basis of their observations, they concluded that sulfur oxidation during disproportionation is not a reversal of the sulfate reduction pathway (Fig. 2B). In agreement with Fuseler et al. (2), we also observed the production of elemental sulfur (measured as S_8_) and polysulfides (S_8_, 0.98 to 3.6 µM; ${\text{S}}_{4}^{2-}$, 15 to 32.3 µM; ${\text{S}}_{5}^{2-}$, 15.9 to 21.7 µM; ${\text{S}}_{6}^{2-}$, 1.72 to 7.68 µM; ${\text{S}}_{7}^{2-}$, 1.00 to 2.43 µM; ${\text{S}}_{8}^{2-}$, 0.61 to 1.14 µM) in the growth medium of *D. alkaliphilus* in the presence of >2 mM molybdate (an inhibitor of sulfate reduction and rDSR-mediated sulfide oxidation). In a culture without molybdate, only polysulfides but no elemental sulfur were found (${\text{S}}_{4}^{2-}$, 0 µM; ${\text{S}}_{5}^{2-}$, 7.31 µM; ${\text{S}}_{6}^{2-}$, 0.27 µM; ${\text{S}}_{7}^{2-}$, 1.02 µM; ${\text{S}}_{8}^{2-}$, 0.29 µM). In the uninoculated control with molybdate, neither elemental sulfur nor polysulfides were found.

10.1128/mBio.00671-17.7 TABLE S2  Comparative genomics of sulfide-sulfur-oxidizing deltaproteobacteria. Shown is the presence/absence of genes putatively involved in the sulfur and nitrogen metabolism of known chemolithotrophic sulfide-oxidizing deltaproteobacteria and *T. dismutans*. Symbols: +, gene present; −, gene absent. Abbreviations: sqr-I, type I SQR; dsr, reductive-type dissimilatory sulfite reductase; HP, hypothetical protein; Mdo, molybdopterin oxidoreductase; Rhd, rhodanese-like gene; nap, periplasmic nitrate reductase. Download TABLE S2, PDF file, 0.1 MB. Copyright © 2017 Thorup et al. 2017 Thorup et al. This content is distributed under the terms of the Creative Commons Attribution 4.0 International license.

Several candidate genes for the initial oxidation of sulfide to elemental sulfur are present in *D. alkaliphilus* (Table S1). Type 1 SQRs catalyze the oxidation of sulfide to sulfur (13); however, the expression level of the *sqr* gene of *D. alkaliphilus* is much lower than that of any other gene encoding sulfur-mediating enzymes (Fig. 1B). Alternatively, the formation of elemental sulfur could be catalyzed by a highly expressed *nrfA* homolog (Fig. 1B), as nitrite reductases are known to also interact with sulfur compounds (14). Lastly, a highly expressed operon containing homologs of genes encoding sulfur-nitrate-transforming enzymes, such as a rhodanese, a molybdopterin oxidoreductase, and polysulfide reductase subunits (Fig. 1B), could be involved in elemental sulfur formation. The lack of all of these genes in *D. thermophilus* and *T. dismutans* (Table S2) may explain the inability of these species to grow by sulfide oxidation and, on the other hand, supports the idea that sulfide oxidation to sulfite by *D. alkaliphilus* and other DSR-utilizing SOMs proceeds via elemental sulfur/polysulfide in a two-step process (2).

It is not clear how the pathway proceeds after the formation of elemental sulfur. The reductive-type sulfite-reductase system in the sulfur bacterium *Allochromatium vinosum* has been shown to be involved in sulfur oxidation (15). On the basis of this, we propose a similar route in *D. alkaliphilus* starting with the formation of DCT by the sulfite reductase DsrMKJOP complex, possibly with the involvement of DsrAB (DSR). The DCT formed would then be oxidized by a reversal of the sulfate reduction pathway (Fig. 2B). Initial oxidation of DCT to sulfite could be performed by either the DSR or a currently unidentified enzyme. In an alternative scenario, DCT could be concurrently reduced back to sulfide by a disproportionation mechanism.

Detailed studies of the various gene products using biochemical characterization and knockouts are needed to ultimately determine the role of Dsr-encoding genes and to fully elucidate the sulfide oxidation pathway in *D. alkaliphilus* and related deltaproteobacteria.

### Reduction of nitrate and nitrite by *D. alkaliphilus*.

Our genomic and transcriptomic data suggest that nitrate reduction to nitrite in *D. alkaliphilus* is catalyzed by a periplasmic nitrate reductase encoded by a *napAGHD* operon (Fig. S1B; Table S3), which is highly expressed during growth by sulfide oxidation but not during nitrate-independent S disproportionation (Fig. 1B and Table S3). The lack of *napB*, which usually encodes the electron donor for catalytic NapA (Fig. S1B), suggests that NapA of *D. alkaliphilus* is monomeric (16), yet its electron donor is unknown; phylogenetically, it would represent a novel type of NapA, unrelated to the NapA proteins of other deltaproteobacteria but similar to that of the bacterial phylum “*Ca*. Kryptonia” (Fig. S4). The electron flow from sulfide to NapA thus remains unresolved.

10.1128/mBio.00671-17.8 TABLE S3  Presence and expression of *D. alkaliphilus* genes involved in nitrogen metabolism. Locus tags for GenBank and the IMG database are listed. Gene expression is shown as RPKM ranks and as log_2_-fold changes (logFC). Abbreviations: sulfur, growth by elemental sulfur disproportionation conditions; nitrate, growth by DNRA-coupled sulfide oxidation. Download TABLE S3, PDF file, 0.2 MB. Copyright © 2017 Thorup et al. 2017 Thorup et al. This content is distributed under the terms of the Creative Commons Attribution 4.0 International license.

10.1128/mBio.00671-17.4 FIG S4  Phylogeny of the *napA* gene of *D. alkaliphilus*. Shown is a maximum-likelihood phylogeny of *napA*-encoded amino acid sequences. Circles represent bootstrap support after 1,000 resamplings as follows: open, ≥70%; filled, ≥90%. The tree was rooted with genes coding for the assimilatory nitrate reductase (*nas*) of *Bacillus subtilis* and for the respiratory nitrate reductase (*narB*) of *Synechococcus elongatus* PCC 7942 (both not shown). Phylogenetic grouping according to Jepson et al. (B. J. Jepson, A. Marietou, S. Mohan, J. A. Cole, C. S. Butler, and D. J. Richardson, Biochem Soc Trans 34:122–126, 2006, https://doi.org/10.1042/BST0340122). Download FIG S4, PDF file, 0.2 MB. Copyright © 2017 Thorup et al. 2017 Thorup et al. This content is distributed under the terms of the Creative Commons Attribution 4.0 International license.

The reduction of nitrite to ammonium by *D. alkaliphilus* is possibly performed by an *nrfA*-encoded periplasmic nitrite reductase (Fig. S1B and Table S3) that is highly expressed during S disproportionation and significantly upregulated during growth by nitrate reduction (Fig. 1B; Table S3). Phylogenetically, NrfA of *D. alkaliphilus* is most similar to that of deltaproteobacterium strain MLMS-1 and distinct from known NrfB- or NrfH-interacting variants (Fig. S5). This is consistent with the lack of *nrfB* and *nrfH* in the genome of *D. alkaliphilus* (Table S3) and the lack of nitrate-induced upregulation of putative *nrfC* and *nrfD* genes (Fig. 1B), which would be essential for a NrfB-associated electron transport chain (17). Finally, a predicted membrane anchor of periplasmic NrfA (Fig. S1B) furthermore suggests that electron flow from sulfide to nitrite in *D. alkaliphilus* is distinct from known DNRA pathways.

10.1128/mBio.00671-17.5 FIG S5  Phylogeny of the *nrfA* gene of *D. alkaliphilus*. Shown is a maximum-likelihood phylogeny of *nrfA*-encoded amino acid sequences. Circles represent bootstrap support after 1,000 resamplings as follows: open, ≥70%; filled, ≥90%. Reference sequences and phylogenetic grouping according to Welsh et al. (A. Welsh, J. C. Chee-Sanford, L. M. Connor, F. E. Löffler, and R. A. Sanford, Appl Environ Microbiol 80:2110–2119, 2014, https://doi.org/10.1128/aem.03443-13). Download FIG S5, PDF file, 0.2 MB. Copyright © 2017 Thorup et al. 2017 Thorup et al. This content is distributed under the terms of the Creative Commons Attribution 4.0 International license.

### Conclusion.

On the basis of our results, we conclude that the deltaproteobacterium *D. alkaliphilus* grows by sulfide oxidation coupled with DNRA and that polysulfide is a likely intermediate, thus supporting the observation reported by Fuseler et al. (2). The high expression level of genes encoding a reductive-type DSR and DsrC under both nitrate-reducing and disproportionating conditions suggests that they are key elements of a sulfide oxidation pathway in *D. alkaliphilus*. However, we could not resolve their specific roles and interactions. Reductive-type *dsrAB* genes are often used as functional markers for SRMs. We conclude that this approach should be used with caution, as our study shows that these genes can also be involved in sulfur disproportionation and sulfide oxidation pathways. Finally, we propose that the close phylogenetic relationship and similar metabolism of *D. alkaliphilus* to the as-yet-uncultured cable bacteria offers the opportunity to establish *D. alkaliphilus* as a tractable model with which to explore the physiology of these intriguing electrogenic bacteria.

### Methods. (i) Cultivation.

*D. alkaliphilus* was grown anaerobically at 30°C in an alkaline mineral medium (5) with sodium carbonate/bicarbonate buffer (0.6 M total Na^+^, pH 9.5), 0.1 M NaCl, 0.5 g liter^−1^ K_2_HPO_4_, and 4 mM NH_4_Cl. After sterilization, the medium was supplemented with 1 mM MgCl_2_ ⋅ 6H_2_O, 10 ml liter^−1^ vitamin solution (18), 1 ml liter^−1^ trace metal SL-10 solution (18), and 1 ml liter^−1^ selenite-tungstate solution (18). For routine growth under sulfide-oxidizing conditions, 3 mM Na_2_S ⋅ 9H_2_O and 3 mM KNO_3_ were added. For growth under sulfur-disproportionating conditions, a pea-sized amount (ca. 1.5 g) of powdered elemental sulfur was added.

### (ii) Determination of reagent concentrations and cell numbers.

Growth by sulfide oxidation with nitrate and the stoichiometry of the conversion were determined in three replicates. Cultures were subsampled every 7 to 8 h for the first 58 h and after 88 h. Subsamples for cell counting were fixed with 4% paraformaldehyde, stained with SYBR Gold (Thermo, Fisher Scientific, Waltham, MA), and quantified by epifluorescence microscopy. Subsamples for sulfide quantification were fixed by the addition of zinc acetate (10% [wt/vol] final concentration) and stored at −20°C. Subsamples for sulfate, ammonium, nitrate, and nitrite quantification were directly frozen to −20°C. Sulfide (methylene blue method) and ammonium (salicylate-hypochlorite method) were measured by spectrophotometry, and sulfate, nitrate, and nitrite were measured by ion chromatography on a Dionex IC 3000 system (Dionex, Sunnyvale, CA).

### (iii) Growth by sulfate reduction.

Growth by sulfate reduction on several common electron donors had been tested previously (5). Additionally, we tested growth by sulfate reduction on hydrogen in 100-ml serum bottles containing 55 ml of mineral medium supplemented with 2 mM Na_2_SO_4_ and a headspace with 3% H_2_. Cultures were incubated for 72 h at 30°C; growth was evaluated by phase-contrast microscopy.

### (iv) Growth by nitrate and nitrite reduction.

Growth on nitrate or nitrite was tested in four replicates at 30°C for 192 h in basic mineral medium supplemented with 6 mM Na_2_S and 10 mM KNO_3_ or KNO_2_. Growth was monitored spectrophotometrically by measurement of optical density at 600 nm.

### (v) Inhibition experiment with sodium molybdate.

Culture medium was supplemented with sodium molybdate from a concentrated stock solution to final concentrations of 2, 5, and 10 mM. The medium was inoculated with a freshly grown culture. Culture-free medium supplemented with sodium molybdate served as a chemical control. Cultures without sodium molybdate served as a biological control. Growth was monitored by microscopy. Polysulfides were quantified via high-performance liquid chromatography (HPLC) following derivatization with methyl triflate (19). Briefly, a 0.1-ml sample, 0.1 ml of phosphate buffer (pH 9.5), and 0.1 ml of methyl triflate were added simultaneously to 0.8 ml of methanol. Concentrations of individual polysulfides with chain lengths of 2 to 8 were determined in the derivatized samples by reversed-phase HPLC with UV detection at 220 and 230 nm and a gradient of methanol and water as the eluent. The method detection limit is 3 to 10 µM, depending upon chain length.

### (vi) Transcriptomic analysis.

*D. alkaliphilus* was grown in triplicate cultures (600 ml each) under sulfur-disproportionating and sulfide-oxidizing conditions and harvested during exponential growth, i.e., after 36 h for sulfide oxidation and after 72 h for sulfur disproportionation. Approximately 5 × 10^7^ cells per sample were collected by filtration on GTTP polycarbonate filters (pore size, 0.22 μm; Millipore) under reduced oxygen concentrations (4 to 19% of atmospheric O_2_) in a glove box. Filters were placed directly into phenol-chloroform-isoamyl alcohol-containing lysis buffer of the kit used for total RNA extraction (Mo Bio RNA PowerSoil Total RNA Isolation kit; Qiagen, Hilden, Germany). Removal of rRNA [Ribo-Zero rRNA removal kit (bacteria); Illumina, San Diego, CA] and transcriptome sequencing (Illumina HiSeq 2500, TruSeq chemistry, SR50 reads) were performed by BaseClear BV (Leiden, Netherlands). Reads (>20 × 10^6^/sample) were mapped onto the genome of *D. alkaliphilus* (GenBank accession number CP001940) by using BBmap version 34.94 (https://sourceforge.net/projects/bbmap/) and default mapping parameters. Mapping data were converted to counts per million (CPM). Pearson correlation coefficients of replicate cultures were between 0.94 and 0.99 on the basis of CPM, indicating good reproducibility across replicates. Highly expressed genes were identified by converting mapping data into reads per kilobase pair per million (RPKM) and subsequent ranking. Differential gene expression was analyzed after normalization and by using the exactTest function as implemented in the R package edgeR version 3.2.4 (20). Gene function prediction is based on the genome annotation provided by the Integrated Microbial Genomes (IMG) database version 4.560 (https://img.jgi.doe.gov/) (genome ID 646564528). Manual confirmation of annotation and, when necessary, reannotation of genes of interest were performed.

### (vii) Phylogenetic analysis.

Sequences for *nrfA* analysis (Table S4) were selected on the basis of a published *nrfA* reference phylogeny (21). Sequences for *napA* analysis (Table S5) were selected on the basis of the published reference phylogeny (16). For *dsrAB* and *dsrC* analyses, only sequences of bona fide SRMs and SOMs were selected as references. Only *dsrC* sequences featuring the two characteristic cysteine residues (22) were selected for analysis. All phylogenies are based on translated nucleotide sequences. *napA*, *nrfA*, and *dsrC* gene sequences were aligned *de novo* by using MAFFT*-*einsi version 7.055b (23) and standard settings. A published reference alignment (10) was used for *dsrAB*. Phylogenies were reconstructed by maximum-likelihood analysis by using RAxML version 8.2.4 (24) with a Γ model of rate heterogeneity and the JTT protein evolution model. Node stability of calculated phylogenies was evaluated by using 1,000 bootstrap replicates.

10.1128/mBio.00671-17.9 TABLE S4  Accession numbers and IMG database (version 4.560; https://img.jgi.doe.gov/) gene identifiers of *nrfA* genes used for phylogenetic analysis. ND, no data (i.e., gene not available in GenBank). Download TABLE S4, PDF file, 0.2 MB. Copyright © 2017 Thorup et al. 2017 Thorup et al. This content is distributed under the terms of the Creative Commons Attribution 4.0 International license.

10.1128/mBio.00671-17.10 TABLE S5  Accession numbers and IMG database (version 4.560; https://img.jgi.doe.gov/) gene identifiers of *napA* genes used for phylogenetic analysis. ND, no data (i.e., gene not available in GenBank or IMG, respectively). Download TABLE S5, PDF file, 0.2 MB. Copyright © 2017 Thorup et al. 2017 Thorup et al. This content is distributed under the terms of the Creative Commons Attribution 4.0 International license.

### (viii) Comparative genomics.

Annotated reference genomes of deltaproteobacterium strain MLMS-1 (IMG genome ID 638341245), *Desulfobulbus elongatus* DSM 2908 (2556921601), *Desulfobulbus japonicus* DSM 18378 (2524614762), *Desulfobulbus mediterraneus* DSM 13871 (2523533605), *Desulfobulbus propionicus* DSM 2032 (649633036), *Desulfocapsa sulfexigens* DSM 10523 (2561511172), *Desulfocapsa thiozymogenes* DSM 7269 (2514885009), and *Desulfotalea psychrophila* LSv54 (637000094) were retrieved from the IMG database (https://img.jgi.doe.gov/). Additional reference genomes of *Dissulfuribacter thermophilus* (accession number MAGO00000000) (12) and *Thermosulfurimonas dismutans* (LWLG00000000) were retrieved from GenBank. Comparative genome analysis was performed with the integrated toolkit for exploration of microbial pangenomes (ITEP) (25) version 1.1 as follows. An ITEP SQL database was generated by using standard cutoff values (BLASTP E-value cutoff, 1E-5; BLASTN E-value cutoff, 1). Genes were clustered on the basis of best bidirectional BLAST hits. Gene clusters were formed with MCL version 12-068 (26) with an inflation value of 2.0 and the maxbit metric.

### Accession number(s).

Transcriptomic sequence data generated in this study have been deposited in the Sequence Read Archive under accession numbers SRS1466493 and SRS1466494.
