# Supplementary material for: Disguised as a Sulfate Reducer: Growth of the Deltaproteobacterium Desulfurivibrio alkaliphilus by Sulfide Oxidation with Nitrate
Source: mBio. 2017 Jul 18;8(4):e00671-17. doi: 10.1128/mBio.00671-17 (PMC5516251; doi:10.1128/mBio.00671-17)
Supplement: FIG S1 [file mbo004173387sf1.pdf]

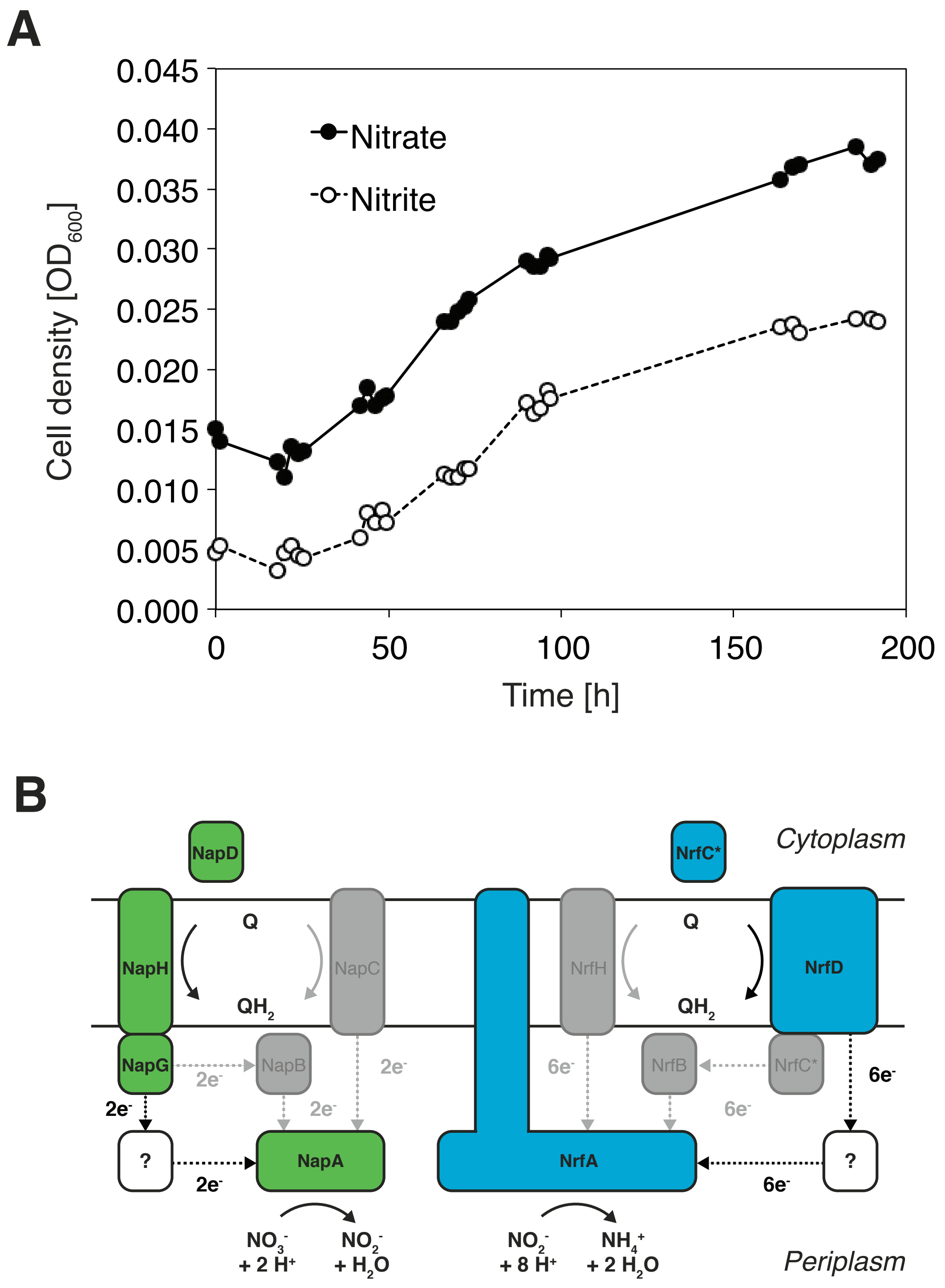

Figure S1. Reduction of nitrate and nitrite by *D. alkaliphilus* (A) Increase of cell density of *D. alkaliphilus* during growth by sulfide oxidation coupled to the reduction of nitrate or nitrite. (B) Model for dissimilatory nitrate reduction to ammonium (DNRA) by *D. alkaliphilus*. The reconstruction is based on genes highly expressed or up-regulated during growth of *D. alkaliphilus* by sulphide-dependent nitrate reduction (see also Table S3). Enzymes previously proposed to be involved in DNRA but whose genes were not detected in *D. alkaliphilus* are shaded in grey. *NrfC: The detected putative *nrf*C gene in the *D. alkaliphilus* genome does not feature the necessary signal peptide for periplasmic localization.
