# Supplementary material for: Disguised as a Sulfate Reducer: Growth of the Deltaproteobacterium Desulfurivibrio alkaliphilus by Sulfide Oxidation with Nitrate
Source: mBio. 2017 Jul 18;8(4):e00671-17. doi: 10.1128/mBio.00671-17 (PMC5516251; doi:10.1128/mBio.00671-17)
Supplement: TABLE S1 [file mbo004173387st1.pdf]

Table S1. Presence and expression of *D. alkaliphilus* genes involved in sulfur metabolism. Locus tags for Genbank and the Integrated Microbial Genomes (IMG) database are listed. Gene expression is shown as RPKM ranks and as log2-fold change (logFC). Abbreviations: Sulfur, growth by elemental sulfur disproportionation conditions; Nitrate, growth by DNRA-coupled sulfide oxidation.

Function	Gene	Locus tag (Genbank)	Locus tag (IMG)	Comment	Gene expression analysis			
					RPKM rank, Sulfur	RPKM rank, Nitrate	logFC (Sulfur/Nitrate)	p-value
ATP sulfurylase	sat	DAAHT2_RS01505	DaAHT2_0293		22	13	-0.75	9.29E-04
Dissimilatory sulfite reduction	dsrA	DAAHT2_RS01520	DaAHT2_0296	part of *dsr*ABD operon	32	22	-0.31	2.36E-01
	dsrB	DAAHT2_RS01525	DaAHT2_0297	part of *dsr*ABD operon	31	26	-0.03	9.28E-01
	dsrD	DAAHT2_RS01530	DaAHT2_0298	part of *dsr*ABD operon	16	8	-0.53	2.87E-02
	dsrC	DAAHT2_RS10360	DaAHT2_2041	genome contains two putative *dsr*C genes	8	1	-1.52	2.17E-10
	dsrM	DAAHT2_RS11670	DaAHT2_2298	part of *dsr*MKJOP operon	225	189	-0.19	3.98E-01
	dsrK	DAAHT2_RS11675	DaAHT2_2299	part of *dsr*MKJOP operon	190	136	-0.45	3.96E-02
	dsrJ	DAAHT2_RS11680	DaAHT2_2300	part of *dsr*MKJOP operon	263	190	-0.34	7.20E-02
	dsrO	DAAHT2_RS11685	DaAHT2_2301	part of *dsr*MKJOP operon	259	211	-0.17	4.24E-01
	dsrP	DAAHT2_RS11690	DaAHT2_2302	part of *dsr*MKJOP operon	205	141	-0.49	3.24E-02
APS reductase	aprA	DAAHT2_RS07525	DaAHT2_1471	part of *apr*BA, *qmo*ABC operon	12	7	-0.24	3.27E-01
	aprB	DAAHT2_RS07530	DaAHT2_1472	part of *apr*BA, *qmo*ABC operon	15	17	0.15	5.65E-01
Quinone-interacting membrane-bound oxidoreductase	qmoA	DAAHT2_RS07520	DaAHT2_1470	part of *apr*BA, *qmo*ABC operon	108	97	-0.36	1.09E-01
	qmoB	DAAHT2_RS07515	DaAHT2_1469	part of *apr*BA, *qmo*ABC operon	143	92	-0.62	6.48E-03
	qmoC	DAAHT2_RS07510	DaAHT2_1468	part of *apr*BA, *qmo*ABC operon	117	101	-0.38	7.85E-02
Sulfate transporter	sulP	*Gene missing*	*Gene missing*					
	sulT	*Gene missing*	*Gene missing*					
Manganese-dependent inorganic pyrophosphatase	ppaC	DAAHT2_RS03525	DaAHT2_0690		80	60	-0.46	4.51E-02
Sulfide:quinone oxidoreductase	sqrA	DAAHT2_RS13475	DaAHT2_2661		1,061	1,499	0.73	1.10E-04
Putative sulfur/nitrate transforming complex	Rhodanese (Rhd)	DAAHT2_RS02120	DaAHT2_0417		21	105	1.77	7.36E-11
	Polysulfide reductase nrfD-like gene	DAAHT2_RS02125	DaAHT2_0418		55	86	0.55	3.68E-02
	Polysulfide reductase nrfC-like gene	DAAHT2_RS02130	DaAHT2_0419		66	81	0.09	7.01E-01
	Molybdopterin oxidoreductase (Mdo)	DAAHT2_RS02135	DaAHT2_0420		61	122	0.73	3.16E-03
	TorD-like nitrate reductase chaperone (torD)	DAAHT2_RS02140	DaAHT2_0421		26	120	1.91	3.20E-09
	Hypothetical protein (Hp)	DAAHT2_RS02145	DaAHT2_0422		1	15	2.00	7.19E-11
