# Supplementary material for: Disguised as a Sulfate Reducer: Growth of the Deltaproteobacterium Desulfurivibrio alkaliphilus by Sulfide Oxidation with Nitrate
Source: mBio. 2017 Jul 18;8(4):e00671-17. doi: 10.1128/mBio.00671-17 (PMC5516251; doi:10.1128/mBio.00671-17)
Supplement: FIG S2 [file mbo004173387sf2.pdf]

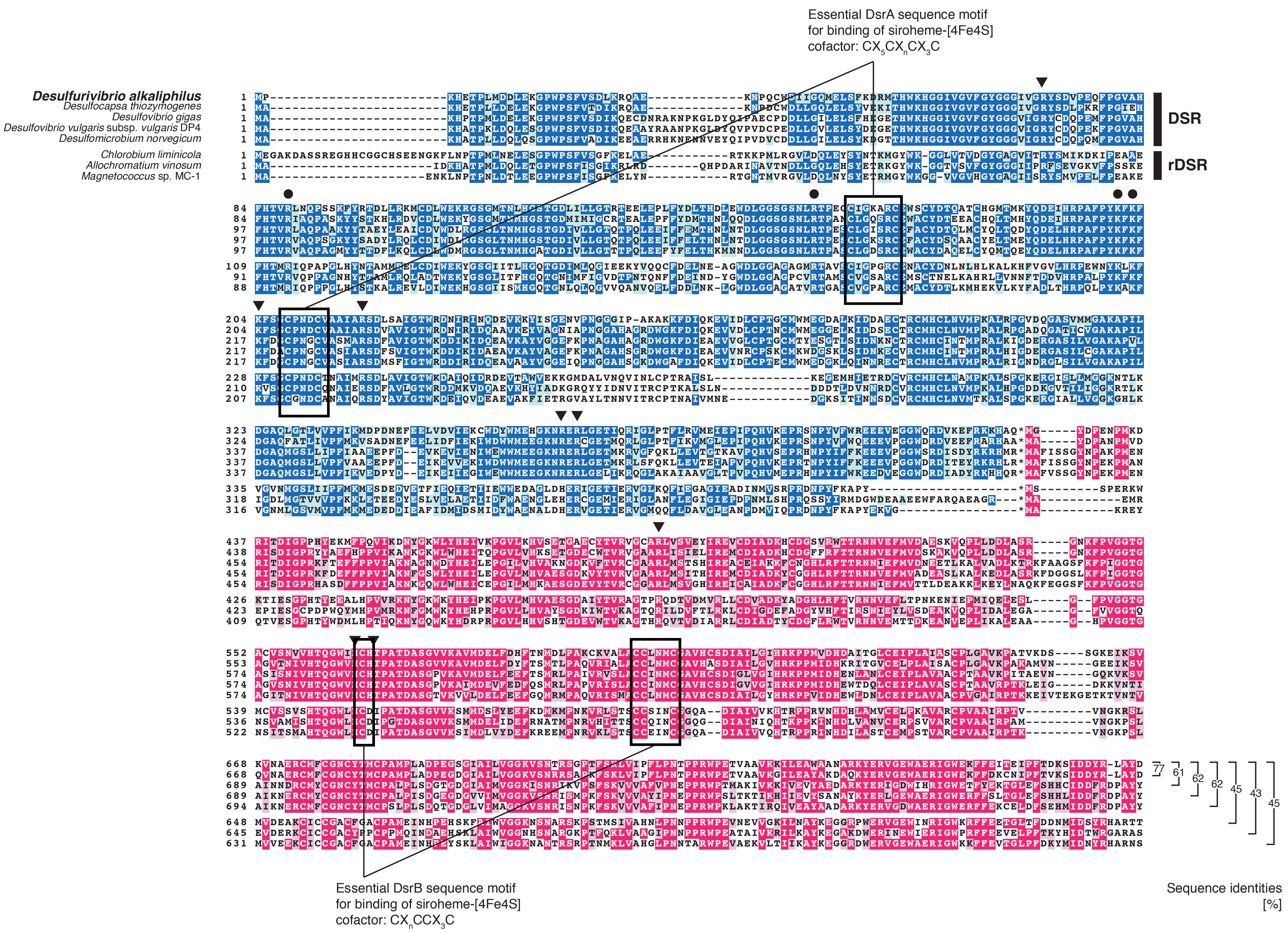
Essential DsrA sequence motif
for binding of siroheme-[4Fe4S]
cofactor: $CX_5CX_nCX_3C$
Desulfurivibrio alkaliphilus
Desulfocapsa thiozymogenes
Desulfovibrio gigas
Desulfovibrio vulgaris subsp. vulgaris DP4
Desulfomicrobium norvegicum
Chlorobium limnicola
Allochromatium vinosum
Magnetococcus sp. MC-1
DSR
rDSR
Essential DsrB sequence motif
for binding of siroheme-[4Fe4S]
cofactor: CX_nCCX_3C
77
61
62
62
45
43
45
Sequence identities
[%]

Figure S2 (previous page). DSR of *Desulfurivibrio alkaliphilus* compared to selected DSRs and rDSRs. Conserved amino acids are shaded with blue (DsrA subunit) or magenta (DsrB). Degree of conservation is indicated with dark (strongly conserved) or light (moderately conserved) shading. Residues involved in sulfite transport and binding (T. F. Oliveira, E. Franklin, J. P. Afonso, A. R. Khan, N. J. Oldham, I. A. C. Pereira, M. Archer, Front Microbiol 2:71,2011, doi: 10.3389/fmicb.2011.00071) are marked: circle, strictly conserved residue; triangle, additionally involved residue. Essential siroheme binding motifs are indicated. The alignment was visualized using BOXSHADE version 3.21; http://sourceforge.net/projects/boxshade).
