# Supplementary material for: Disguised as a Sulfate Reducer: Growth of the Deltaproteobacterium Desulfurivibrio alkaliphilus by Sulfide Oxidation with Nitrate
Source: mBio. 2017 Jul 18;8(4):e00671-17. doi: 10.1128/mBio.00671-17 (PMC5516251; doi:10.1128/mBio.00671-17)
Supplement: FIG S3 [file mbo004173387sf3.pdf]

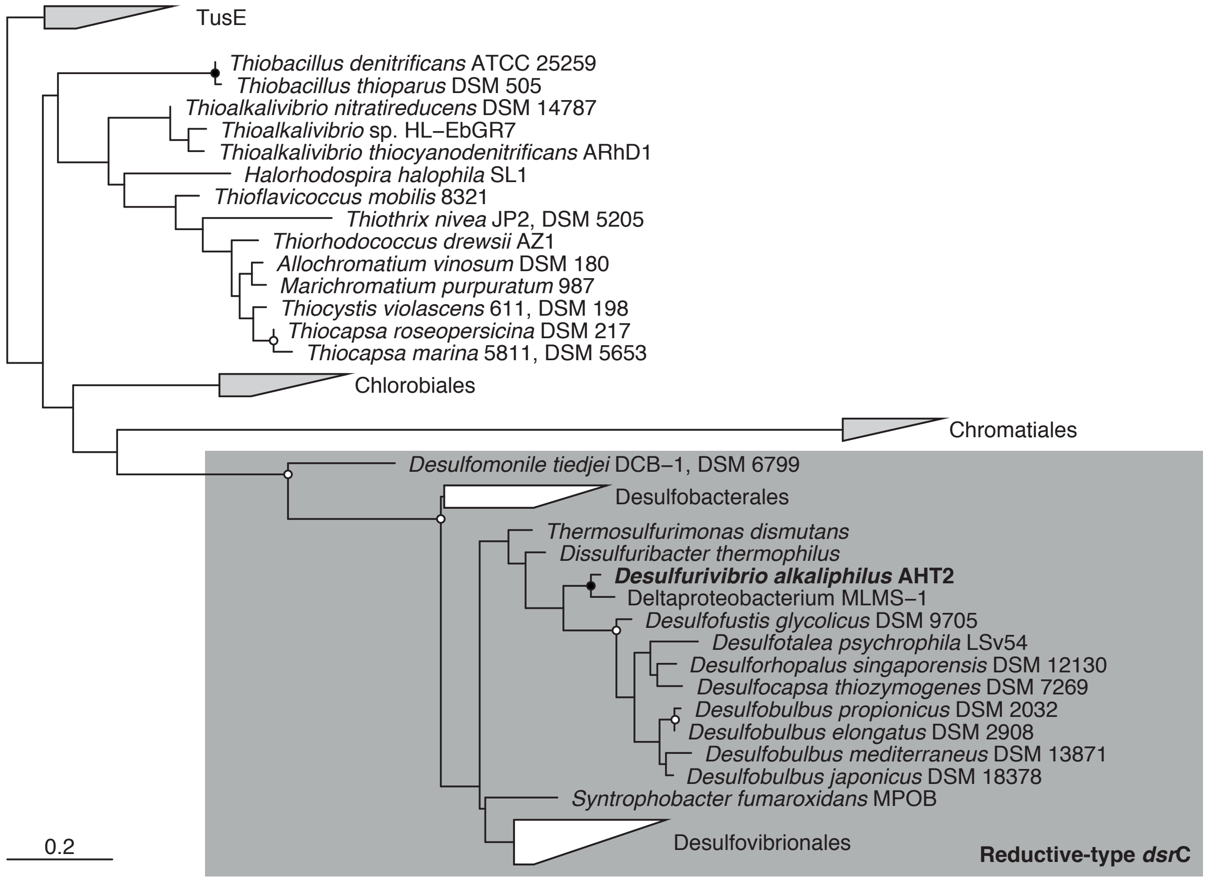

Figure S3. Phylogeny of the *dsr*C gene of *D. alkaliphilus*. Shown is a maximum likelihood phylogeny of *dsr*C genes of D. alkaliphilus and known sulfate-reducing as well as sulfide-oxidizing bacteria. Circles represent bootstrap support after 1,000 re-samplings: open, ≥70%; filled, ≥90%.
