# Supplementary material for: Disguised as a Sulfate Reducer: Growth of the Deltaproteobacterium Desulfurivibrio alkaliphilus by Sulfide Oxidation with Nitrate
Source: mBio. 2017 Jul 18;8(4):e00671-17. doi: 10.1128/mBio.00671-17 (PMC5516251; doi:10.1128/mBio.00671-17)
Supplement: TABLE S2 [file mbo004173387st2.pdf]

Table S2. Comparative genomics of sulfide/sulfur-oxidizing Deltaproteobacteria**.** Shown is the presence/absence of genes putatively involved in the sulfur and nitrogen metabolism of known chemolithotrophical sulfide-oxidizing Deltaproteobacteria and *Thermosulfurimonas dismutans*. Symbols: +, gene present; -, gene absent. Abbreviations: sqr-I, type I sulfide:quinone oxidoreductase; dsr, reductive-type dissimilatory sulfite reductase; apr, adenosine-5'-phosphosulfate reductase; HP, hypothetical protein; Mdo, molybdopterin oxidoreductase; Rhd, Rhodanese-like gene; nap, periplasmic nitrate reductase.

Species	e-donor	e-acceptor	Sulfur metabolism																						Nitrogen metabolism				
			sqr-I	*dsr*									*apr*		*sat*	*qmo*			polysulfide-nrf operon						*nrf*A	*nap*			
				A	B	C	D	M	K	J	O	P	A	B		A	B	C	Hp	*tor*D-like	Mdo	*nrf*C-like	*nrf*D-like	Rhd		A	D	G	H
Desulfurivibrio alkaliphilus	Sulfide	Nitrate/Nitrite	+	+	+	+	+	+	+	+	+	+	+	+	+	+	+	+	+	+	+	+	+	+	+	+	+	+	+
Deltaproteobacterium MLMS-1	Sulfide	Arsenate	+	+	+	+	+	+	+	+	+	+	+	+	+	+	+	+	+	+	+	+	+	+	+	-	-	-	-
Dissulfibacter thermophilus	Sulfur	Nitrate	-	+	+	+	+	+	+	+	+	+	+	+	+	+	+	+	-	-	-	+	+	-	-	+	+	+	+
Thermosulfurimonas dismutans	Sulfur	Nitrate	-	+	+	+	+	+	+	+	+	+	+	+	+	+	+	+	-	-	-	-	+	-	-	+	+	+	+
