# Supplementary material for: Disguised as a Sulfate Reducer: Growth of the Deltaproteobacterium Desulfurivibrio alkaliphilus by Sulfide Oxidation with Nitrate
Source: mBio. 2017 Jul 18;8(4):e00671-17. doi: 10.1128/mBio.00671-17 (PMC5516251; doi:10.1128/mBio.00671-17)
Supplement: FIG S4 [file mbo004173387sf4.pdf]

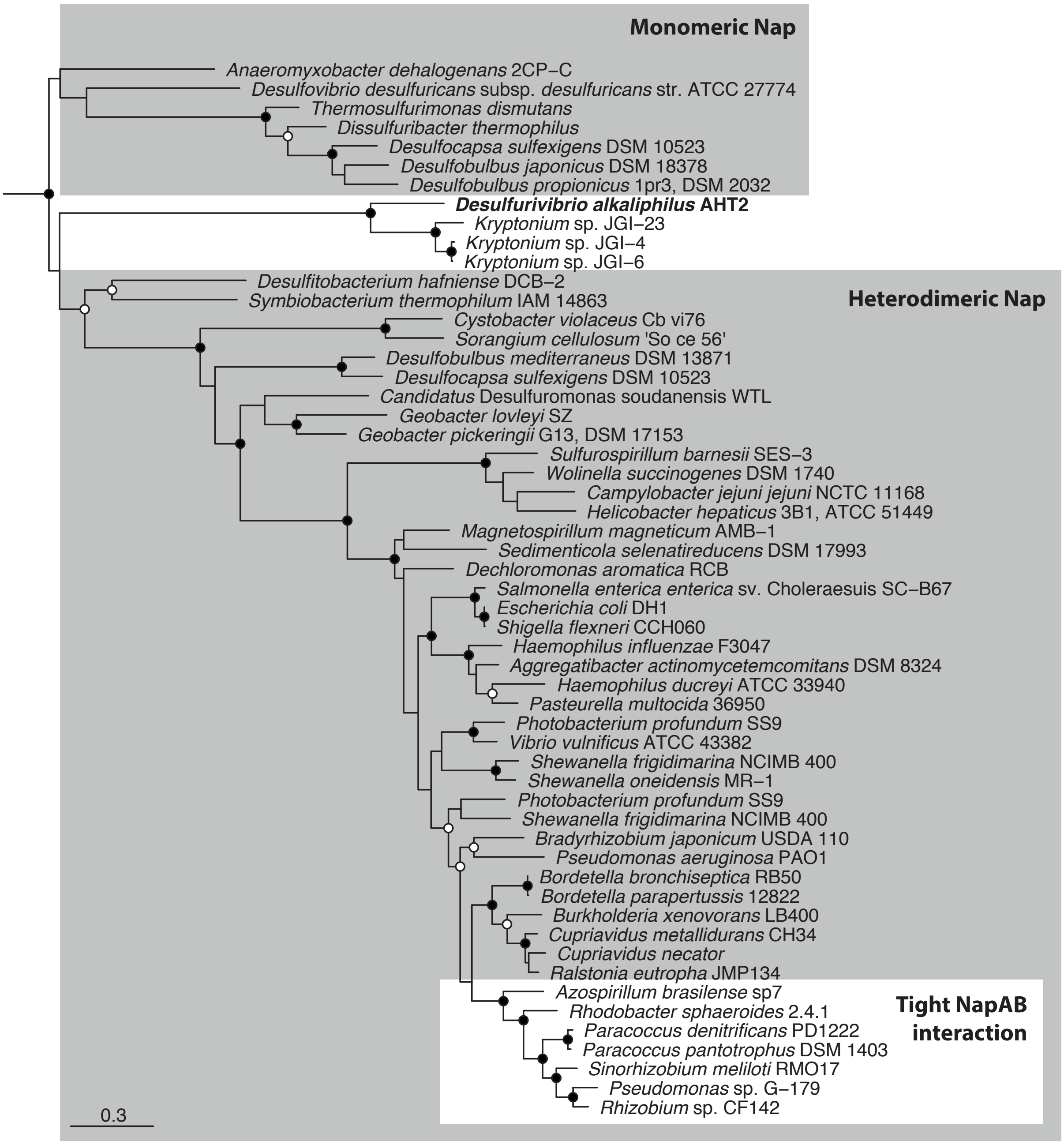

Figure S4. Phylogeny of the *nap*A gene of *D. alkaliphilus*. Maximum likelihood (ML) phylogeny of *nap*A amino acid sequences. Circles represent bootstrap support after 1,000 re-samplings: open, ≥70%; filled, ≥90%. The tree was rooted with genes coding for the assimilatory nitrate reductase (*nas*) of *Bacillus subtilis* and for the respiratory nitrate reductase (*nar*B) of *Synechococcus elongatus* PCC 7942 (both not shown). Phylogenetic grouping according to Jepson et al. [Biochem Soc Trans 34(1):122-126, 2006, doi:10.1042/BST0340122].
