# Supplementary material for: Disguised as a Sulfate Reducer: Growth of the Deltaproteobacterium Desulfurivibrio alkaliphilus by Sulfide Oxidation with Nitrate
Source: mBio. 2017 Jul 18;8(4):e00671-17. doi: 10.1128/mBio.00671-17 (PMC5516251; doi:10.1128/mBio.00671-17)
Supplement: FIG S5 [file mbo004173387sf5.pdf]

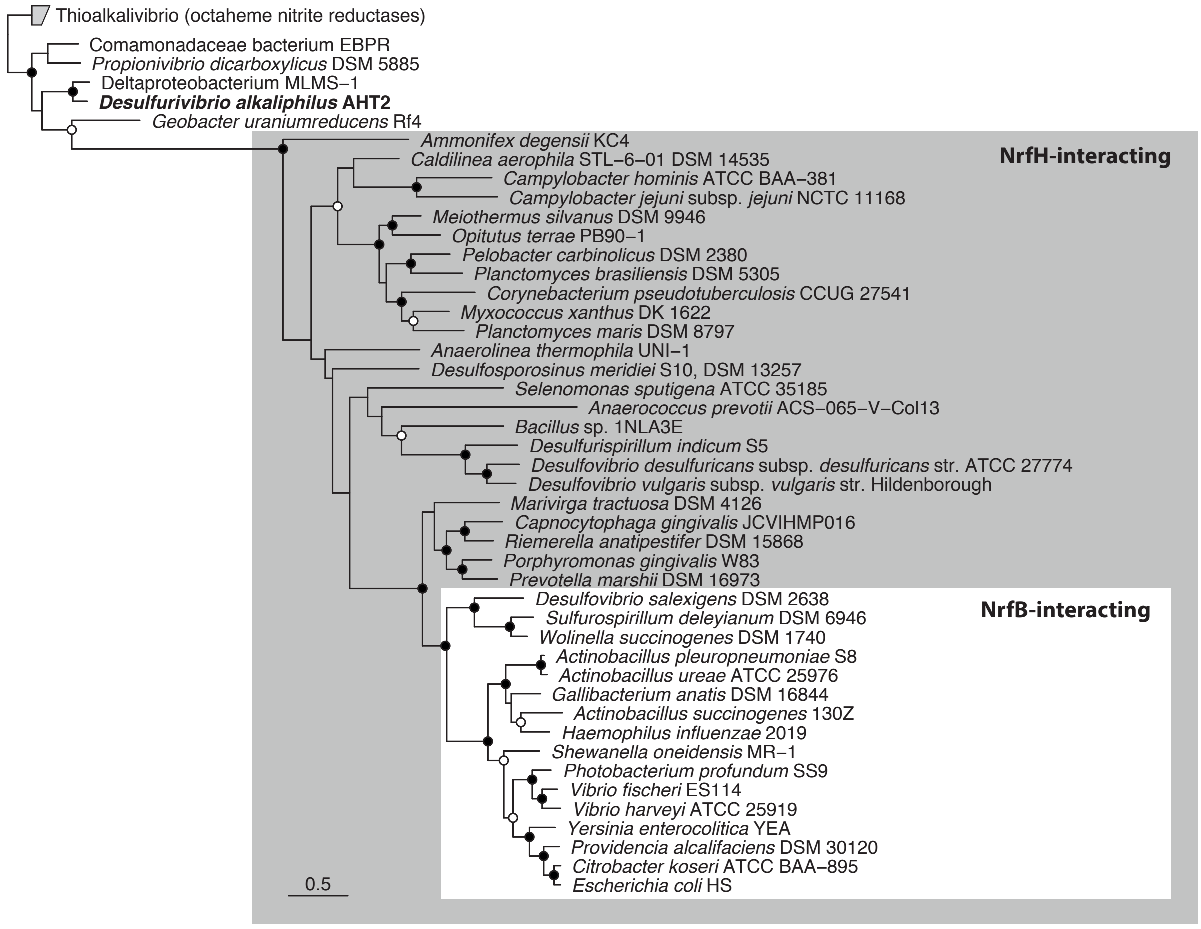

Figure S5. Phylogeny of the *nrf*A gene of *D. alkaliphilus*. Maximum likelihood (ML) phylogeny of *nrf*A amino acid sequences. Circles represent bootstrap support after 1,000 re-samplings: open, ≥70%; filled, ≥90%. Reference sequences and phylogenetic grouping according to Welsh et al. [Appl Environ Microbiol 80(7):2110-2119, 2014, doi: 10.1128/aem.03443-13].
