# Supplementary material for: Disguised as a Sulfate Reducer: Growth of the Deltaproteobacterium Desulfurivibrio alkaliphilus by Sulfide Oxidation with Nitrate
Source: mBio. 2017 Jul 18;8(4):e00671-17. doi: 10.1128/mBio.00671-17 (PMC5516251; doi:10.1128/mBio.00671-17)
Supplement: TABLE S4 [file mbo004173387st4.pdf]

Table S4. Accession numbers and Integrated Microbial Genomes (IMG) database (version 4.560; https://img.jgi.doe.gov/) gene identifiers of *nrf*A genes used for phylogenetic analysis. n.d.: no data, i.e. gene is not available at Genbank.

Species name	Accession number	IMG gene identifier
Actinobacillus pleuropneumoniae S8	n.d.	2552278133
Actinobacillus succinogenes 130Z	NC 009655	640807619
Actinobacillus ureae ATCC 25976	NZ AEVG01000127	650340686
Ammonifex degensii KC4	NC 013385	646359879
Anaerococcus prevotii ACS-065-V-Col13	AEXM01000028	2529738369
Anaerolinea thermophila UNI-1	NC 014960	649906650
Bacillus sp. 1NLA3E	n.d.	2506744287
Caldilinea aerophila STL-6-01, DSM 14535	n.d.	2513225030
Campylobacter hominis ATCC BAA-381	NC 009714	640869848
Campylobacter jejuni subsp. *jejuni* NCTC 11168	NC 002163	637040891
Capnocytophaga gingivalis JCVIHMP016	NZ ACLQ01000018	644450706
Citrobacter koseri ATCC BAA-895	NC 009792	640917778
Comamonadaceae bacterium EBPR	n.d.	2619975620
Corynebacterium pseudotuberculosis CCUG 27541	n.d.	2628297476
Deltaproteobacterium MLMS-1	NZ AAQF01000083	639155534
Desulfosporosinus meridiei S10, DSM 13257	n.d.	2510242216
Desulfovibrio desulfuricans subsp. *desulfuricans* str. ATCC 27774	NC 011883	643580866
Desulfovibrio salexigens DSM 2638	NC 012881	644838886
Desulfovibrio vulgaris subsp. *vulgaris* str. Hildenborough	NC 002937	637121843
Desulfurispirillum indicum S5	NC 014836	649844489
Desulfurivibrio alkaliphilus AHT2	NC 014216	646845286
Escherichia coli HS	NC 009800	640923413
Gallibacterium anatis DSM 16844	n.d.	2514917813
Geobacter uraniumreducens Rf4	NC 009483	640551483
Haemophilus influenzae 2019	n.d.	2630849089
Marivirga tractuosa DSM 4126	NC 014759	649787403
Meiothermus silvanus DSM 9946	NC 014212	646843608
Myxococcus xanthus DK 1622	NC 008095	638023578
Opitutus terrae PB90-1	NC 010571	641694276
Pelobacter carbinolicus DSM 2380	NC 007498	637752476
Photobacterium profundum SS9	NC 006370	637585967
Planctomyces brasiliensis DSM 5305	NC 015174	649980400
Planctomyces maris DSM 8797	NZ ABCE01000055	641112668
Porphyromonas gingivalis W83	NC 002950	637150428
Prevotella marshii DSM 16973	NZ AEEI01000055	648809557
Propionivibrio dicarboxylicus DSM 5885	n.d.	2599430875
Providencia alcalifaciens DSM 30120	NZ ABXW01000073	643148102
Riemerella anatipestifer DSM 15868	NC 014738	649778058
Selenomonas sputigena ATCC 35185	NZ ACKP02000048	646081185
Shewanella oneidensis MR-1	NC 004347	637345732
Sulfurospirillum deleyianum DSM 6946	NC 013512	646423959
Thioalkalivibrio nitratireducens DSM 14787	CP003989	2521963319
Thioalkalivibrio paradoxus ARh 1	n.d.	2513007479
Thioalkalivibrio thiocyanoxidans ARh 4	n.d.	2506728454
Vibrio fischeri ES114	NC 006840	637636232
Vibrio harveyi ATCC 25919	n.d.	2583678091
Wolinella succinogenes DSM 1740	NC 005090	637456212
Yersinia enterocolitica YEA	n.d.	2611555138
