# Supplementary material for: Disguised as a Sulfate Reducer: Growth of the Deltaproteobacterium Desulfurivibrio alkaliphilus by Sulfide Oxidation with Nitrate
Source: mBio. 2017 Jul 18;8(4):e00671-17. doi: 10.1128/mBio.00671-17 (PMC5516251; doi:10.1128/mBio.00671-17)
Supplement: TABLE S5 [file mbo004173387st5.pdf]

Table S5. Accession numbers and Integrated Microbial Genomes (IMG) database (version 4.560; https://img.jgi.doe.gov/) gene identifiers of *nap*A genes used for phylogenetic analysis. n.d.: no data, i.e. gene is not available at Genbank or IMG, respectively.

**Species name**	**Accession number**	**IMG gene identifier**
Aggregatibacter actinomycetemcomitans DSM 8324	n.d.	2515250942
Azospirillum brasilense sp7 - copy 1	n.d.	2599104047
Azospirillum brasilense sp7 - copy 2	n.d.	2599101664
Bordetella bronchiseptica RB50	n.d.	2607649690
Bordetella parapertussis 12822	n.d.	2607390835
Bradyrhizobium japonicum USDA 110	n.d.	2606847837
Burkholderia xenovorans LB400	n.d.	2607180546
Campylobacter jejuni jejuni NCTC 11168	n.d.	2608345870
Candidatus Desulfuromonas soudanensis WTL	n.d.	2609285564
Cupriavidus metallidurans CH34	n.d.	2607208611
Cupriavidus necator	WP_011154126	640427642
Cystobacter violaceus Cb vi76	KFA92432	2592593039
Desulfobulbus mediterraneus DSM 13871	n.d.	2523913625
Desulfocapsa sulfexigens DSM 10523	AGF79815	2562337019
Desulfurivibrio alkaliphilus AHT2	WP_013164110.1	646847134
Escherichia coli DH1	ACX39123	646935360
Geobacter lovleyi SZ	ACD94779	642676451
Geobacter pickeringii G13, DSM 17153	n.d.	2609280171
Haemophilus ducreyi ATCC 33940	n.d.	2599173274
Haemophilus influenzae F3047	CBY86593	649868883
Helicobacter hepaticus 3B1, ATCC 51449	n.d.	2607801258
Kryptonium sp. JGI-23	n.d.	2601849902
Kryptonium sp. JGI-4	n.d.	2599799712
Kryptonium sp. JGI-6	n.d.	2600397245
Magnetospirillum magneticum AMB-1	n.d.	2608300189
Paracoccus denitrificans PD1222	WP_011750941	639772025
Pasteurella multocida 36950	AET16720	2512391891
Photobacterium profundum SS9 - copy 1	n.d.	2608100144
Photobacterium profundum SS9 - copy 2	n.d.	2608098895
Pseudomonas aeruginosa PAO1	n.d.	2607363311
Pseudomonas sp. G-179	AAD46689	n.d.
Ralstonia eutropha JMP134	n.d.	2608280881
Rhizobium sp. CF142	EJJ28402	2511196655
Rhodobacter sphaeroides 2.4.1	YP_345332	640069465
Salmonella enterica enterica sv. Choleraesuis SC-B67	n.d.	2607616886
Salmonella enterica enterica sv. Typhimurium LT2	n.d.	2607860623
Shewanella oneidensis MR-1	n.d.	2606813272
Shigella flexneri CCH060	EIQ09873	2531700739
Sinorhizobium meliloti RMO17	n.d.	2598504540
Sorangium cellulosum 'So ce 56'	CAN94865	641348992
Sulfurospirillum barnesii SES-3	AFL68099	2507135060
Wolinella succinogenes DSM 1740	n.d.	2607821073
