# Supplementary material for: Disguised as a Sulfate Reducer: Growth of the Deltaproteobacterium Desulfurivibrio alkaliphilus by Sulfide Oxidation with Nitrate
Source: mBio. 2017 Jul 18;8(4):e00671-17. doi: 10.1128/mBio.00671-17 (PMC5516251; doi:10.1128/mBio.00671-17)
Supplement: TABLE S3 [file mbo004173387st3.pdf]

Table S3. Presence and expression of *D. alkaliphilus* genes involved in nitrogen metabolism. Locus tags for Genbank and the Integrated Microbial Genomes (IMG) database are listed. Gene expression is shown as RPKM ranks and as log2-fold change (logFC). Abbreviations: Sulfur, growth by elemental sulfur disproportionation conditions; Nitrate, growth by DNRA-coupled sulfide oxidation.

**Function**	**Gene**	**Locus tag (Genbank)**	**Locus tag (IMG)**	**Comment**	**Gene expression analysis**			
					**RPKM rank, Sulfur**	**RPKM rank Nitrate**	**logFC (Sulfur/Nitrate)**	**p-value**
Periplasmic dissimilatory nitrate reduction	NapA	DAAHT2_RS09680	DaAHT2_1907	Part of *nap*AGHD operon	937	151	-2.53	3.58E-23
	NapB	*Gene missing*	*Gene missing*					
	NapC	*Gene missing*	*Gene missing*					
	NapD	DAAHT2_RS09695	DaAHT2_1910	Part of *nap*AGHD operon	1149	302	-1.89	4.37E-21
	NapG	DAAHT2_RS13960	DaAHT2_1908	Part of *nap*AGHD operon; Originally called *nap*G gene does not contain required signal peptide for periplasmic localization.	2140	659	-2.49	6.63E-38
	NapH	DAAHT2_RS09690	DaAHT2_1909	Part of *nap*AGHD operon	2115	520	-2.75	1.14E-32
	NapM	*Gene missing*	*Gene missing*					
Cytoplasmic dissimilatory nitrate reduction	NarG	*Gene missing*	*Gene missing*					
	NarH	*Gene missing*	*Gene missing*					
	NarI	*Gene missing*	*Gene missing*					
	NarJ	*Gene missing*	*Gene missing*					
Dissmilatory nitrite reduction to ammonium (DNRA)	Periplasmic nitrite reductase (NrfA/ONR)	DAAHT2_RS00360	DaAHT2_0065	Unlike other NrfA/ONR has its own transmemebrane helix	30	5	-1.69	1.32E-11
	NrH	*Gene missing*	*Gene missing*					
	NrfC	DAAHT2_RS02130	DaAHT2_0419	Only putative NrfC; signla peptide for periplasmic localization missing	66	81	0.09	7.01E-01
	NrfD	DAAHT2_RS02125	DaAHT2_0418	Only putative NrfD	55	86	0.55	3.68E-02
Reverse hydroxylamine:ubiquinone reductase module (HURM) pathway	Hydroxylamine oxidoreductase (HaoA)	*Gene missing*	*Gene missing*	Locus in *N. profundicola*: NAMH 1280				
	Tetra-heme cytochrome c (CycB)	*Gene missing*	*Gene missing*	Locus in *N. profundicola*: NAMH 0559				
	Hydroxylamine reductase (Hcp/Har)	DAAHT2_RS11870	DaAHT2_2339	Locus in *N. profundicola*: NAMH 1074	183	986	2.45	4.24E-25
	Fe-S containing protein	*Gene missing*	*Gene missing*	Locus in *N. profundicola*: NAMH 1302				
	Hydroxylamine transporter (AmtB-like)	DAAHT2_RS00680	DaAHT2_0128	Locus in *N. profundicola*: NAMH 0215; NAMH 0397	2,568	2,542	0.07	7.96E-01
	Glutamine synthetase	*Gene missing*	*Gene missing*	Locus in *N. profundicola*: NAMH 0501				
	Glutamate synthase (NADPH), small chain	*Gene missing*	*Gene missing*	Locus in *N. profundicola*: NAMH 1054				
	Glutamate synthase (NADPH), large chain	*Gene missing*	*Gene missing*	Locus in *N. profundicola*: NAMH 1569				
